# Risk Factors for HIV-1 seroconversion among Taiwanese men visiting gay saunas who have sex with men

**DOI:** 10.1186/1471-2334-11-334

**Published:** 2011-12-05

**Authors:** Yen-Ju Chen, Yu-Ting Lin, Marcelo Chen, Szu-Wei Huang, Su-Fen Lai, Wing-Wai Wong, Hung-Chin Tsai, Yu-Huei Lin, Hsin-Fu Liu, Shu-Yu Lyu, Yi-Ming A Chen

**Affiliations:** 1 AIDS Prevention and Research Center, National Yang-Ming University, Taipei, Taiwan; 2 Department of Urology, Mackay Memorial Hospital, Taipei, Taiwan; 3 Mackay Medicine, Nursing and Management College, New Taipei City, Taiwan; 4 Institute of Microbiology and Immunology, National Yang-Ming University, Taipei, Taiwan; 5 Taipei Veterans' General Hospital, Taipei, Taiwan; 6 Kaohsiung Veterans' General Hospital, Kaohsiung, Taiwan; 7 Taichung Veterans' General Hospital, Taichung, Taiwan; 8 Department of Medical Research, Mackay Memorial Hospital, Taipei, Taiwan; 9 School of Public Health, Taipei Medical University, Taipei, Taiwan

## Abstract

**Background:**

Men having sex with men (MSM) accounts for 33.6% of all reported cases of HIV-1 infection in Taiwan. The aim of this study was to investigate the epidemiology of HIV-1 infection among MSM in gay saunas in Taiwan.

**Methods:**

Patrons of 5 gay saunas were recruited for a weekly volunteer counseling and testing program from 2001 to 2005. Questionnaires were collected for a risk factor analysis. HIV-1 subtypes were determined using DNA sequencing and phylogenetic analyses.

**Results:**

HIV-1 prevalence rates among MSM in gay saunas in 2001 through 2005 were 3.4%, 5.1%, 8.9%, 8.5%, and 8.3%, respectively. In total, 81 of 1, 093 (7.4%) MSM had HIV-1 infection. Fifty-two HIV-1 strains were genotyped, and all of them were subtype B. HIV-seropositive men were significantly younger than the seronegatives. Only 37.1% used condoms every time during sexual intercourse. A multivariate logistic regression analysis showed that the risk factors for HIV-1 were being uncircumcised (odds ratio (OR) = 2.19; 95% confidence interval (CI), 1.08~4.45); having sexual intercourse with at least 2 partners during each sauna visit (≥ 2 vs. ≤ 1, OR = 1.71; 95% CI, 1.02~2.89); and the role played during anal intercourse (versatile vs. an exclusively insertive role, OR = 2.76; 95% CI, 1.42~5.36).

**Conclusions:**

Overall, 7.4% Taiwanese MSM participating in this study had HIV-1 subtype B infection. Uncircumcised, being versatile role during anal intercourse, and having sex with more than one person during each sauna visit were main risk factors for HIV-1 infection.

## Background

Since the global outbreak of HIV/AIDS, HIV has been transmitted among different vulnerable populations. According to a recent review, the global trend of HIV-1 infection among men having sex with men (MSM) has continued to increase, especially in East Asia, Africa, and Russia [1]. As of September 2011, there were 22, 335 known cases of HIV infection in Taiwan, and Taiwanese nationals accounted for 96.5% of those cases. Of the Taiwanese nationals, 92.5% were male. Populations at risk for HIV infection include injection drug users (30.4%), homosexual men (38.3%), heterosexual men (21.6%), and bisexual men (8.1%). Therefore, MSM are a high risk group for HIV infection in Taiwan [2].

Since 1996~1997, the use of highly active antiretroviral therapy (HAART) has significantly decreased HIV/AIDS-related mortality [3]. It has been reported that unprotected sex has increased among MSM after the introduction of HAART, which has resulted in dramatic decreases in the morbidity and mortality from HIV-1 infection [4]. Taiwan began to provide free HAART to all HIV-1/AIDS patients in April 1997 [5,6]. Although successful HAART can reduce the contagiousness of HIV-1, the fact that HIV-1 transmission still occurs in the ART-era indicates that transmission occurs from undiagnosed, untreated, or unsuccessfully treated patients [7].

Risks factors for HIV-1 infection in MSM include unprotected anal intercourse [8-10], multiple sexual partners [11], a history of drug use [12-14], a history of commercial sexual transactions [11,15], and a history of other sexually transmitted diseases (STDs) [1]. In Taiwan, homosexuals usually gather in parks, gay bars, and gay saunas, and because gay saunas provide the opportunity for sexual intercourse, individuals who frequent saunas are at a high risk of contracting STDs. Therefore, gay saunas are an ideal place for voluntary counseling and testing for HIV-1 infection [16]. The aims of this study were (1) to determine the prevalence and trends of HIV-1 infection among MSM frequenting gay saunas from 2001 to 2005; (2) to identify the risk factors associated with HIV-1 infection; and (3) to determine the viral subtypes of HIV-1 strains found in MSM in Taiwan.

## Methods

### Participants

Before beginning the study, we contacted gay saunas in Taipei, Hsinchu, and Taichung to inquire as to their willingness to cooperate in participant recruitment. Anonymous HIV testing and education were provided during peak hours on weekends. Blood samples were obtained by venipuncture in private rooms. A single participant was interviewed at a time.

Gay saunas in Taipei were visited every other weekend, while those in Hsinchu and Taichung were visited once a month. All recruited participants provided blood samples, and they could choose whether or not to complete a questionnaire. This research study was approved by the Institutional Review Board of National Yang-Ming University. Before testing for HIV-1/2 antibodies, researchers provided the necessary counseling. Test results were available 2 weeks after the blood samples were obtained. Participants with negative results were encouraged to undergo follow-up screening every 6 months, while participants with positive results were initially counseled by a professional social worker and referred to a hospital for further counseling. Positive blood samples then underwent HIV-1 subtyping.

### HIV-1 Screening, Subtyping, and Phylogenetic Analysis

Participants provided 5~10 ml of peripheral blood for HIV-1 antibody testing and subtyping. Recombinant HIV-1/HIV-2 EIA (Murex Diagnostics Limited, Dartford, UK) was used for HIV screening. Positive samples were reconfirmed by HIV Western blot 2.2 (Genelabs, Singapore). A nested polymerase chain reaction (PCR) was used to determine the *gag *and *env *subtypes [17]. Sequence alignments with different subtype reference sequences from the Los Alamos HIV database (available at: http://www.hiv.lanl.gov) were conducted using the BioEdit program (vers. 7.0.9) [18]. Phylogenetic trees were constructed using the Neighbor-joining (NJ) and maximum-likelihood (ML) methods using the MEGA program (vers. 5.0) [19]. The General Time Reversible (GTR)+G+I substitution model was the best fitting model as tested using MEGA5 Bayesian information criterion (BIC) scores, the corrected Akaike information criterion (AIC) value, and the maximum-likelihood value (*lnL*). The gamma distribution shape parameter (G) was 1.0342. The robustness of the phylogenetic trees was statistically evaluated by a bootstrap analysis with 1000 bootstrap samples.

### Statistical Analysis

Pearson's χ^2 ^test and Fisher's exact test were performed in a univariate analysis to investigate differences in demographic data and consumer behavior patterns among MSM. Student's *t*-test or an analysis of variance (ANOVA) was conducted to examine age differences. A multivariate logistic regression analysis was performed to identify risk factors associated with HIV-1 infection.

### Nucleotide Sequence Accession Numbers

HIV-1 *env *sequences obtained in this study were deposited in GenBank with the accession numbers JN631752~JN631791.

## Results

Between 2001 and 2005, 1, 093 participants (81 HIV-1 positive and 1, 012 HIV-1 negative) participated in this study. The positive rates of HIV-1 and syphilis were 7.4% and 7.6%, respectively. A trend analysis showed that HIV-1 positive rates significantly increased from 3.4% in 2001 to 6.1% in 2002, and to 8.9% in 2003 (*p *= 0.031). Then, it reached a plateau at 8.5% in 2004 and 8.4% in 2005. In contrast, 2 peaks syphilis infection rates were seen in 2002 and 2005 (Table 1).

**Table 1:** Prevalence of HIV-1 and syphilis among men having sex with men (MSM) from gay saunas in Taiwan in 2001~2005

	HIV-1 prevalence		Syphilis prevalence	
Study year	*N*	(%)	*n*	(%)
2001	3/88	(3.4)	7/88	(8.0)
2002	12/236	(5.1)	22/236	(9.3)
2003	28/316	(8.9)	21/316	(6.6)
2004	21/248	(8.5)	11/248	(4.4)
2005	17/205	(8.3)	22/205	(10.7)
Total	81/1093	(7.4)	83/1093	(7.6)

The mean age of participants was 32.4 (SD, 8.1) years, with a range of 17~81 years. Among participants, 54.87% had a high school degree, and 91.7% were single (Additional file 1). Significant differences were found between participants who brought their own condoms to the sauna every visit (79.5% HIV-1 positive vs. 66.9% HIV-1 negative, *p *< 0.05). Among those who did not bring their own condoms, more HIV-1-positive participants than their counterparts purchased condoms from the counter (53.3% vs. 39.1%, *p *< 0.05) (Additional file 2).

HIV-1-positive participants had sexual intercourse with more people in a 3-month period on average than their counterparts (4.8 ± 5.1 vs. 4.0 ± 6.4, *p *< 0.05). More HIV-1-negative than HIV-1-positive participants had insertive roles (35.9% vs. 19.5%). Overall, 27% of study participants were circumcised, including 16.5% HIV-1-positive and 26.8% HIV-1-negative participants (*p *= 0.045) (Additional file 3). The major reasons for testing or not were shown in Additional file 4. Nineteen percent of the study participants had an STD history (Additional file 5).

According to univariate logistic regression, several factors were associated with HIV-1 infection, they included: those with versatile roles (OR = 2.55, 95% CI = 1.41~4.61), those performing anal intercourse (OR = 2.73, 95% CI = 1.08~6.86), those performing anal rimming (OR = 2.03, 95% CI = 1.24~3.34), those having sex with at least 2 partners per visit (OR = 1.73, 95% CI = 1.08~2.80), being uncircumcised (OR = 1.85, 95% CI = 1.01~3.42), and those aged under 35 years (OR = 2.70, 95% CI = 1.07~6.79), as shown in Table 2.

**Table 2:** Univariate logistic regression analysis of risk factors in HIV-1-infected men having sex with men (MSM)

Variable	HIV (+) *N *= 81 *n *(%)	HIV (-) *N *= 1, 012 *n *(%)	Odds ratio	95% Confidence interval
**Role during anal intercourse**				
Versatile	53/77 (68.8)	410/824 (49.8)	2.55	1.41~4.61
Exclusively receptive	9/77 (11.7)	118/824 (14.3)	1.51	0.64~3.53
Exclusively insertive	15/77 (19.5)	296/824 (35.9)	1	
**Frequency of anal sex during sexual intercourse**				
Always/frequently/occasionally/rarely*	73/78 (93.6)	793/941 (84.3)	2.73	1.08~6.86
Never	5/78 (6.4)	148/941 (15.7)	1	
**Number of sexual partners in the past 3 months**				
≥ 2	55/69 (79.7)	565/802 (70.4)	1.65	0.90~3.02
0 or 1	14/69 (20.3)	237/802 (29.6)	1	
**Frequency of anal rimming**				
Always/frequently/occasionally/rarely*	54/78 (69.2)	493/938 (52.6)	2.03	1.24~3.34
Never	24/78 (30.8)	445/938 (47.4)	1	
**Number of sexual partners during each sauna visit**				
≥ 2	39/74 (52.7)	316/808 (39.1)	1.73	1.08~2.80
0 or 1	35/74 (47.3)	492/808 (60.9)	1	
**Frequency of sauna visits**				
Once a month or less	54/67 (80.6)	665/815 (81.5)	0.94	0.50~1.77
More than once a month	13/67 (19.4)	151/816 (18.5)	1	
**Other STDs**				
Yes	19/77 (24.6)	172/926 (18.6)	1.44	0.83~2.47
No	58/77 (75.3)	754/926 (81.4)	1	
**Recreational drugs before visiting sauna**				
Always/frequently/occasionally/rarely*	11/78 (14.1)	76/946 (8.0)	1.88	0.95~3.71
Never	67/78 (85.9)	870/946 (92.0)	1	
**Frequency of condom use during sexual intercourse**				
Occasionally/Rarely/Never*	34/61 (55.7)	308/652 (47.2)	1.39	0.82~2.36
Always	27/61 (44.3)	344/652 (52.8)	1	
**Circumcised**				
No	66/79 (83.5)	692/945 (73.2)	1.85	1.01~3.42
Yes	13/79 (16.5)	253/945 (26.8)	1	
**Age**				
≤ 35 years	68/78 (87.2)	637/923 (69.0)	2.70	1.07~6.79
> 35 years	10/78 (12.8)	286/923 (31.0)	1	

* Detailed information is available in the additional file 3.

Results from stepwise multivariate logistic regression analysis indicated that those with versatile roles (OR = 2.76, 95% CI = 1.42~5.36), those had sexual intercourse with at least 2 men during each visit (OR = 1.71, 95% CI = 1.02~2.89), being uncircumcised (OR = 2.19, 95% CI = 1.08~4.45) were related to HIV-1 infection (Table 3).

**Table 3:** Stepwise multivariate logistic regression analysis of risk factors in HIV-1-infected men having sex with men

Variable	HIV (+) *N *= 81 *n *(%)	HIV (-) *N *= 1, 012 *n *(%)	Odds ratio	95% Confidence interval
**Role during anal intercourse**				
Versatile	53/77 (68.8)	410/724 (49.8)	2.76	1.42~5.36
Exclusively receptive	9/77 (11.7)	118/724 (14.3)	1.44	0.57~3.66
Exclusively insertive	15/77 (19.5)	296/724 (35.9)	1	
**Number of sexual partners during each sauna visit**				
≥ 2	39/74 (52.7)	316/808 (39.1)	1.71	1.02~2.89
0 or 1	35/74 (47.3)	492/808 (60.9)	1	
**Circumcised**				
No	66/79 (83.5)	692/945 (73.2)	2.19	1.08~4.45
Yes	13/79 (16.5)	253/945 (26.8)	1	

Consistent tree topologies were observed in both the NJ and ML trees. Analysis of the *env *subtype in 52 HIV-1-positive participants showed that all participants had subtype B (Additional file 6). Results showed that some HIV-1 strains from gay saunas and Venereal Disease Control Institute (VDCI) clustered with bootstrap values of > 85%.

## Discussion

According to the Centers for Disease Control, in 2009, the HIV-positive rate based on screening at major hospitals, blood centers, military bases, and prisons was 54.96/100, 000 (1651/3, 003, 735) [2]. In this study, we screened 1, 093 participants and found a syphilis-positive rate of 7.6% and a HIV-1-positive rate of 7.4%, which were significantly higher than those reported by the Centers for Disease Control.

From a physiological point of view, the strong diffusion capabilities of the rectum facilitate HIV infection [20]. Analysis by the number of sexual partners showed a direct correlation between the number of sexual partners and the probability of HIV infection [21]. In addition, condom use can significantly decrease propagation of the HIV virus. In this study, over 80% of study participants had anal intercourse, which was comparable to the 71.4% reported by Ko *et al*. in 1997 [21]. The percentage of participants wore a condom every time and for the entire duration of sexual intercourse (Additional file 3) was similar to that reported by Ko *et al*. [21], suggesting that through the years, the proportion of homosexuals performing anal intercourse has remained unchanged and the proportion of condom use has remained low.

Frequent visits to saunas increase the probability of contracting STDs [16]. Participants in our study had a higher rate of STDs, and participants with STDs are more susceptible to HIV infection (Additional file 5). The route of STD transmission is a risk factor for HIV infection and that STDs increase the HIV infection rate 2~5-fold [22]. A stepwise multiple regression analysis revealed that role played during anal intercourse, the number of sexual partners during each sauna visit, and the circumcision status were risk factors associated with HIV-1 infection in MSM frequenting gay saunas. These were also found to be risk factors in other countries [16,22].

Male circumcision is a religious commandment in Judaism and Islam. The world prevalence of men with circumcision is 12.5%~33%, and is especially high in the USA, Canada, and Africa and among Islamic people [23]. Approximately 80% of US men are circumcised [24], in which white males had the highest prevalence (> 70%) and Hispanics had the lowest prevalence (≤ 40%) [25]. Ko *et al*. reported that the prevalence of circumcision in Taiwan was 7.2%~8.7% [26]. In this study, our findings indicated that circumcision of MSM provides a certain degree of protection against HIV-1 infection. Previous studies showed that male circumcision reduced the likelihood of female-to-male transmission of HIV-1 infection by 50%~60% [27-29]. Concerning the protective effect of circumcision for MSM, several studies reported that circumcision had a significant protective effect [30-33]. In contrast, other studies reported that circumcised MSM had greater chances for contracting HIV infection although they were statistically nonsignificant [34,35]. Since Buddhism and Taoism are the two main religions in Taiwan (44.5% and 24.1%, respectively), while Christianity and Islam populations are relatively small (10% and 1%, respectively) [36], religion might not have been a confounder in this study. Recently, an emerging epidemic among Taiwanese MSM was revealed when the number of new HIV-infected homosexuals increased from 571 (17% of all new HIV-infected) in 2005 to 1, 275 (71%) in 2010 [37]. Circumcision may be one of the approaches that should be adapted to control the epidemic of HIV-1 infection among Taiwanese MSM in the future.

HIV screening among high-risk group is cost-effective. Unlinked anonymous testing can provide high-risk individuals with a secure means of testing and can prevent a selection bias [38]. In this study, we performed purposive sampling and snowballing in order to increase the sample size [39]. However, because the sampling was not random, results from this study cannot be used to represent the prevalence of HIV infection in gay sauna patrons or of the general MSM population across Taiwan. In addition, because the screening was anonymous, most participants used aliases, and only a few participants agreed to be followed-up. This hindered our follow-up efforts and impeded us from estimating the incidence in a cohort study. The seropositive rate in this study was high. We therefore suggest that future studies expand the sampling area and survey other places where MSM congregate so as to estimate the size of the MSM population and thereby fully monitor trends of the HIV virus in this high-risk population.

## Conclusions

Of MSM participating in this study, 7.4% (81/1093) were infected with HIV-1, and all of them had subtype B. The circumcision status, the role played during anal intercourse, and the number of sexual partners during each sauna visit were the three main risk factors for HIV-1 infection.

This study accessed gay saunas to obtain a deeper understanding of the sexual behavior of MSM in Taiwan. Results from this study can serve as a reference for researchers interested in these behavioral patterns and for local authorities promoting health education among MSM.

## Competing interests

The authors declare that they have no competing interests.

## Authors' contributions

SFL, YTL, and YJC collected samples and data, performed the statistical analysis, and drafted the manuscript. MC helped draft the final manuscript. SWH and HFL carried out the sequence alignment and phylogenetic analyses. WWW, HCT, and YHL participated in the design of the study. YMC and SYL conceived of the study, participated in its design and coordination, and helped draft and revised the manuscript. All authors read and approved the final manuscript.

## Pre-publication history

The pre-publication history for this paper can be accessed here:

http://www.biomedcentral.com/1471-2334/11/334/prepub

## Supplementary Material

Additional file 1 **Demographic data of HIV-1 positive and negative MSM from gay saunas**. Click here for file

Additional file 2 **Consumer behavior of MSM in gay saunas**. Click here for file

Additional file 3 **Sexual behavioral patterns and HIV-associated risk factors of MSM**. Click here for file

Additional file 4 **Reasons for screening**. Click here for file

Additional file 5 **STD-associated symptoms and treatment status in MSM**. Click here for file

Additional file 6 **Phylogenetic analysis of HIV-1 strains among Taiwanese men having sex with men (MSM) who visited gay saunas**. The maximum-likelihood tree was constructed with the MEGA program (vers. 5.0) using the *env *nucleotide sequence from different HIV-1 strains. Bootstrap values (1000 bootstrap samples) are indicated beside the branches in percent. HIV-1 strains from the gay saunas (circle symbol) and the Venereal Disease Control Institute (VDCI) (triangle symbol) were labeled and characterized to denote the patients' characteristics, including the year of diagnosis, from 2001 to 2003; the sex of the patient (M, male); the risk factor of the patient (Ho, homosexual; He, heterosexual; Bi, bisexual); and the location of the gay saunas (N, north; M, central). The scale bar indicates the evolutionary distance. Click here for file
