# Supplementary material for: Risk Factors for HIV-1 seroconversion among Taiwanese men visiting gay saunas who have sex with men
Source: BMC Infect Dis. 2011 Dec 5;11:334. doi: 10.1186/1471-2334-11-334 (PMC3295735; doi:10.1186/1471-2334-11-334)
Supplement: Additional file 1 — Demographic data of HIV-1 positive and negative MSM from gay saunas. [file 1471-2334-11-334-S1.DOC]

**Additional file 1** - Demographic data of HIV-1 positive and negative MSM from gay saunas

| Variable | HIV (+) | | HIV (-) | | Total | | p-value^〒^ |
| --- | --- | --- | --- | --- | --- | --- | --- |
|  | *N*=81 (%)  *n* (%) | | *N*=1,012 (%)  *n* (%) | | *N*=1,093 (%)  *n* (%) | |  |
| **Age** |  |  |  |  |  |  | 0.036 |
| ≤19 | 1/78 | (1.2) | 19/923 | (2.1) | 20/1001 | (2.0) |  |
| 20-29 | 40/78 | (51.3) | 335/923 | (36.3) | 375/1001 | (37.5) |  |
| 30-39 | 32/78 | (41.0) | 403/923 | (43.7) | 435/1001 | (43.5) |  |
| 40-49 | 4/78 | (5.1) | 133/923 | (14.4) | 137/1001 | (13.7) |  |
| ≥50 | 1/78 | (1.3) | 33/923 | (3.6) | 34/1001 | (3.4) |  |
| Mean±SD | 30.0±6.1 | | 32.6±8.3 | | 32.4±8.1 | | <0.001^†^ |
| (Range) | (18-50) | | (17-81) | | (17-81) | |  |
| **Education level** |  |  |  |  |  |  | 0.303 |
| Elementary school | 1/78 | (1.3) | 13/929 | (1.4) | 14/1007 | (1.4) |  |
| Middle school | 1/78 | (1.3) | 22/929 | (2.4) | 23/1007 | (2.3) |  |
| High school | 50/78 | (64.1) | 502/929 | (54.0) | 552/1007 | (54.8) |  |
| University | 25/78 | (32.1) | 337/929 | (36.3) | 362/1007 | (35.9) |  |
| Graduate school | 1/78 | (1.3) | 55/929 | (5.9) | 56/1007 | (5.6) |  |
| **Marital status** |  |  |  |  |  |  | 0.417 |
| Single | 76/78 | (97.4) | 838/919 | (91.2) | 914/997 | (91.7) |  |
| Married | 2/78 | (2.6) | 59/919 | (6.4) | 61/997 | (6.1) |  |
| Divorced | 0/78 | (0.0) | 14/919 | (1.5) | 14/997 | (1.4) |  |
| Separated | 0/78 | (0.0) | 7/919 | (0.8) | 7/997 | (0.7) |  |
| Widowed | 0/78 | (0.0) | 1/919 | (0.1) | 1/997 | (0.1) |  |

^〒^. Pearson Chi-Square. ^†^. Student T Test
