# Supplementary material for: Risk Factors for HIV-1 seroconversion among Taiwanese men visiting gay saunas who have sex with men
Source: BMC Infect Dis. 2011 Dec 5;11:334. doi: 10.1186/1471-2334-11-334 (PMC3295735; doi:10.1186/1471-2334-11-334)
Supplement: Additional file 2 — Consumer behavior of MSM in gay saunas. [file 1471-2334-11-334-S2.DOC]

**Additional file 2** - Consumer behavior of MSM in gay saunas

| Variable | HIV (+) | | HIV (-) | | | Total | | | p-value^〒^ |
| --- | --- | --- | --- | --- | --- | --- | --- | --- | --- |
|  | *N*=81 (%)  *n* (%) | | *N*=1,012 (%)  *n* (%) | | | *N*=1,093 (%)  *n* (%) | | |  |
| **Average time between each visit** | | |  |  | |  | |  | 0.820 |
| ≦7 days | 12/67 | (17.9) | 158/816 | (19.4) | | 170/883 | | (19.3) |  |
| 8-14 days | 15/67 | (22.4) | 154/816 | (18.9) | | 169/883 | | (19.1) |  |
| 15-21 days | 13/67 | (19.4) | 136/816 | (16.7) | | 149/883 | | (16.9) |  |
| 22-30 days | 14/67 | (20.9) | 217/816 | (26.6) | | 231/883 | | (26.2) |  |
| >30 days | 13/67 | (19.4) | 151/816 | (18.5) | | 164/883 | | (18.6) |  |
| Mean ± SD | 28.9±32.1 | | 35.6±63.1 | | | 35.1±61.3 | | | <0.001^†^ |
| (Range) | (1-180) | | (1-730) | | | (1-730) | | |  |
| **Average number of sexual partners during each sauna visit** | | | | | | |  | | 0.030 |
| 0 | 1/74 | (1.4) | 71/808 | (8.8) | | 72/882 | (8.2) | |  |
| 1~2 | 60/74 | (81.1) | 647/808 | (80.1) | | 707/882 | (80.2) | |  |
| >3 | 13/74 | (17.6) | 90/808 | (11.1) | | 103/882 | (11.7) | |  |
| Mean±SD | 1.8±1.2 | | 1.5±1.2 | | | 1.5±1.2 | | | 0.351 |
| (Range) | (0-8) | | (0-13) | | | (0-13) | | |  |
| **Visited saunas in other countries** | | |  | |  |  |  | | 0.433^‡^ |
| Yes | 20/73 | (27.4) | 271/834 | | (32.5) | 291/907 | | (32.1) |  |
| **Drank alcohol before visited sauna** | | |  | |  |  | |  | 0.710 |
| Always | 0/78 | (0.0) | 9/951 | | (0.9) | 9/1029 | | (0.9) |  |
| Frequently | 3/78 | (3.8) | 23/951 | | (2.4) | 26/1029 | | (2.5) |  |
| Occasionally | 7/78 | (9.0) | 93/951 | | (9.8) | 100/1029 | | (9.7) |  |
| Rarely | 15/78 | (19.2) | 147/951 | | (15.5) | 162/1029 | | (15.7) |  |
| Never | 53/78 | (67.9) | 679/951 | | (71.4) | 732/1029 | | (71.1) |  |
| **Took drugs (amphetamines, stimulants, ecstasy) before visiting sauna** | | | | | | | | | 0.290 |
| Always | 0/78 | (0.0) | 2/946 | | (0.2) | 2/1024 | | (0.2) |  |
| Frequently | 1/78 | (1.3) | 3/946 | | (0.3) | 4/1024 | | (0.4) |  |
| Occasionally | 4/78 | (5.1) | 24/946 | | (2.5) | 28/1024 | | (2.7) |  |
| Rarely | 6/78 | (7.7) | 47/946 | | (5.0) | 53/1024 | | (5.2) |  |
| Never | 67/78 | (85.9) | 870/946 | | (92.0) | 937/1024 | | (91.5) |  |
| **Brought own condoms to sauna** | | |  | |  |  | |  | 0.038 |
| Always | 27/78 | (34.6) | 356/969 | | (36.7) | 383/1047 | | (36.6) |  |
| Frequently | 18/78 | (23.1) | 131/969 | | (13.5) | 149/1047 | | (14.2) |  |
| Occasionally | 17/78 | (21.8) | 161/969 | | (16.6) | 178/1047 | | (17.0) |  |
| Rarely | 7/78 | (9.0) | 101/969 | | (10.4) | 108/1047 | | (10.3) |  |
| Never | 9/78 | (11.5) | 220/969 | | (22.7) | 229/1047 | | (21.9) |  |
| **Bought condoms from sauna counter** | | |  | |  |  | |  | 0.017 |
| Always | 11/77 | (14.3) | 133/959 | | (13.2) | 144/1036 | | (13.9) |  |
| Frequently | 7/77 | (9.1) | 95/959 | | (9.4) | 102/1036 | | (9.8) |  |
| Occasionally | 23/77 | (29.9) | 178/959 | | (16.5) | 201/1036 | | (19.4) |  |
| Rarely | 17/77 | (22.1) | 148/959 | | (16.6) | 165/1036 | | (15.9) |  |
| Never | 19/77 | (24.7) | 405/959 | | (44.2) | 424/1036 | | (40.9) |  |

^〒^. Pearson Chi-Square. ^†^. Student T Test. ^‡^. Fisher's Exact Test
