# Supplementary material for: Risk Factors for HIV-1 seroconversion among Taiwanese men visiting gay saunas who have sex with men
Source: BMC Infect Dis. 2011 Dec 5;11:334. doi: 10.1186/1471-2334-11-334 (PMC3295735; doi:10.1186/1471-2334-11-334)
Supplement: Additional file 3 — Sexual behavioral patterns and HIV-associated risk factors of MSM. [file 1471-2334-11-334-S3.DOC]

**Additional file 3** - Sexual behavioral patterns and HIV-associated risk factors of MSM

| Variable | HIV (+) | | HIV (-) | | | Total | | | p-value^〒^ |
| --- | --- | --- | --- | --- | --- | --- | --- | --- | --- |
|  | *N*=81 (%)  *n* (%) | | *N*=1,012 (%)  *n* (%) | | | *N*=1,093 (%)  *n* (%) | | |  |
| **Number of sexual partners in the past 3 months** | | | | | |  |  | | 0.147 |
| 0 | 5/69 | (7.2) | 39/802 | (4.9) | | 44/871 | (5.1) | |  |
| 1-2 | 21/69 | (30.4) | 360/802 | (44.9) | | 381/871 | (43.7) | |  |
| 3-4 | 18/69 | (26.1) | 179/802 | (22.3) | | 197/871 | (22.6) | |  |
| 5-6 | 11/69 | (15.9) | 121/802 | (15.1) | | 132/871 | (15.2) | |  |
| >6 | 14/69 | (20.3) | 103/802 | (12.8) | | 117/871 | (13.4) | |  |
| Mean±SD | 4.8±5.1 | | 4.0±6.4 | | | 4.0±6.3 | | | 0.020^†^ |
| (Range) | (0-30) | | (0-100) | | | (0-100) | | |  |
| **Frequency of anal sex during sexual intercourse** | | | | | |  | |  | 0.162 |
| Always | 2/78 | (2.6) | 21/941 | | (2.2) | 23/1019 | | (2.3) |  |
| Frequently | 13/78 | (16.7) | 131/941 | | (13.9) | 144/1019 | | (14.1) |  |
| Occasionally | 33/78 | (42.3) | 312/941 | | (33.2) | 345/1019 | | (33.9) |  |
| Rarely | 25/78 | (32.1) | 329/941 | | (35.0) | 354/1019 | | (34.7) |  |
| Never | 5/78 | (6.4) | 148/941 | | (15.7) | 153/1019 | | (15.0) |  |
| **Role during anal sex** | |  |  | |  |  | |  | 0.016 |
| Exclusively receptive | 9/77 | (11.7) | 118/824 | | (14.3) | 127/901 | | (14.1) |  |
| Exclusively insertive | 15/77 | (19.5) | 296/824 | | (35.9) | 311/901 | | (34.5) |  |
| Mostly receptive | 32/77 | (41.6) | 224/824 | | (27.2) | 256/901 | | (28.4) |  |
| Mostly insertive | 13/77 | (16.9) | 128/824 | | (15.5) | 141/901 | | (15.6) |  |
| Versatile | 8/77 | (10.4) | 58/824 | | (7.0) | 66/901 | | (7.3) |  |
| **Frequency of anal rimming during sexual intercourse** | | | | | |  | |  | 0.015 |
| Always | 2/78 | (2.6) | 7/938 | | (0.7) | 9/1016 | | (0.9) |  |
| Frequently | 4/78 | (5.1) | 36/938 | | (3.8) | 40/1016 | | (3.9) |  |
| Occasionally | 11/78 | (14.1) | 146/938 | | (15.6) | 157/1016 | | (15.5) |  |
| Rarely | 37/78 | (47.4) | 304/938 | | (32.4) | 341/1016 | | (33.6) |  |
| Never | 24/78 | (30.8) | 445/938 | | (47.4) | 469/1016 | | (46.2) |  |
| **Fixed sexual partner over the past 3 months** | | | | |  |  | |  | 0.096^‡^ |
| Yes | 42/80 | (52.5) | 613/989 | | (62.0) | 655/1069 | | (61.3) |  |
| **Commercial sexual transaction** | | |  | |  |  | |  | 0.508^‡^ |
| Yes | 9/78 | (11.5) | 143/934 | | (15.3) | 152/1012 | | (15.0) |  |
| **Place of sexual transaction (multiple answers allowed)** | | | | | |  | |  |  |
| Taiwan | 5/9 | (55.6) | 82/143 | | (57.3) | 87/151 | | (57.6) | 0.911^‡^ |
| Abroad | 5/9 | (55.6) | 69/141 | | (48.9) | 74/150 | | (49.3) | 0.717^‡^ |
| **Gender of partner in sexual transaction** | | | | |  |  | |  |  |
| Male | 9/9 | (100.0) | 113/140 | | (80.7) | 122/149 | | (81.9) | 0.156^‡^ |
| Female | 0/9 | (0.0) | 32/140 | | (22.9) | 32/149 | | (21.5) | 0.106^‡^ |
| Transsexual | 0/9 | (0.0) | 1/140 | | (0.7) | 1/149 | | (0.7) | 0.940^‡^ |
| Circumscribed |  |  |  | |  |  | |  | 0.045^‡^ |
| Yes | 13/79 | (16.5) | 253/945 | | (26.8) | 266/1024 | | (27.0) |  |
| **Redundant prepuce in uncircumscribed men** | | | | |  |  | |  | 0.245^‡^ |
| Yes | 17/55 | (30.9) | 126/542 | | (23.2) | 143/597 | | (24.0) |  |
| **Frequency of condom use during sexual intercourse** | | | | | |  | |  | 0.065 |
| Always | 27/79 | (34.2) | 344/921 | | (37.4) | 371/1000 | | (37.1) |  |
| Frequently | 18/79 | (22.8) | 269/921 | | (29.2) | 287/1000 | | (28.7) |  |
| Occasionally | 24/79 | (30.4) | 158/921 | | (17.2) | 182/1000 | | (18.2) |  |
| Rarely | 6/79 | (7.6) | 91/921 | | (9.9) | 97/1000 | | (9.7) |  |
| Never | 4/79 | (5.1) | 59/921 | | (6.4) | 63/1000 | | (6.3) |  |
| **Requested condom use during sexual intercourse** | | | | | |  | |  | 0.025 |
| Always | 15/73 | (20.5) | 206/798 | | (21.7) | 221/871 | | (21.2) |  |
| Frequently | 26/73 | (35.6) | 162/798 | | (18.3) | 188/871 | | (19.1) |  |
| Occasionally | 12/73 | (16.4) | 152/798 | | (19.5) | 164/871 | | (19.5) |  |
| Rarely | 11/73 | (15.1) | 106/798 | | (13.6) | 117/871 | | (14.0) |  |
| Never | 9/73 | (12.3) | 172/798 | | (26.9) | 181/871 | | (26.3) |  |

^〒^. Pearson Chi-Square. ^†^. Student T Test. ^‡^. Fisher's Exact Test
