# Supplementary material for: Risk Factors for HIV-1 seroconversion among Taiwanese men visiting gay saunas who have sex with men
Source: BMC Infect Dis. 2011 Dec 5;11:334. doi: 10.1186/1471-2334-11-334 (PMC3295735; doi:10.1186/1471-2334-11-334)
Supplement: Additional file 4 — Reasons for screening. [file 1471-2334-11-334-S4.DOC]

**Additional file 4 - Reasons for screening**

| Variable | HIV (+) | | HIV (-) | | Total | | p-value^〒^ |
| --- | --- | --- | --- | --- | --- | --- | --- |
|  | *N*=81 (%)  *n* (%) | | *N*=1,012 (%)  *n* (%) | | *N*=1,093 (%)  *n* (%) | |  |
| **Previous screening** |  |  |  |  |  |  | 0.025^‡^ |
| Yes | 46/81 | (56.8) | 691/995 | (69.4) | 737/1076 | (68.5) |  |
| **Reasons for previous screening (multiple answers allowed)** | | | | |  |  |  |
| Health exam | 30/44 | (68.1) | 411/665 | (61.8) | 441/709 | (62.2) | 0.398 |
| Worried about possible infection | 8/44 | (18.2) | 140/665 | (21.1) | 148/709 | (20.9) | 0.650 |
| Worried about possible sexual partner infection | 2/44 | (4.5) | 27/664 | (4.1) | 29/708 | (4.1) | 0.817^‡^ |
| Infected with other STD | 1/44 | (2.3) | 10/664 | (1.5) | 11/708 | (1.6) | 0.654^‡^ |
| Blood donation | 1/44 | (2.3) | 25/664 | (3.8) | 26/708 | (3.7) | 0.693^‡^ |
| Other | 9/44 | (20.5) | 100/664 | (15.1) | 109/708 | (15.4) | 0.337 |
| **Reasons for no previous screening** | | |  |  |  |  | 0.628 |
| Worried about knowing results | 6/34 | (17.6) | 47/280 | (16.8) | 53/314 | (16.9) |  |
| Not feeling at risk for HIV infection | 2/34 | (5.9) | 37/280 | (13.2) | 39/314 | (12.4) |  |
| Concerned about confidentiality | 6/34 | (17.6) | 33/280 | (11.8) | 39/314 | (12.4) |  |
| Never thought about testing | 10/34 | (29.4) | 84/280 | (30.0) | 94/314 | (29.9) |  |
| Inconvenient | 8/34 | (23.5) | 49/280 | (17.5) | 57/314 | (18.2) |  |
| Other | 2/34 | (5.9) | 30/280 | (10.7) | 32/314 | (10.2) |  |

^〒^. Pearson Chi-Square. ^‡^. Fisher's Exact Test
