# Supplementary material for: Risk Factors for HIV-1 seroconversion among Taiwanese men visiting gay saunas who have sex with men
Source: BMC Infect Dis. 2011 Dec 5;11:334. doi: 10.1186/1471-2334-11-334 (PMC3295735; doi:10.1186/1471-2334-11-334)
Supplement: Additional file 5 — STD-associated symptoms and treatment status in MSM. [file 1471-2334-11-334-S5.DOC]

**Additional file 5 - STD-associated symptoms and treatment status in MSM**

| Variable | HIV (+) | | HIV (-) | | Total | | p-value^〒^ |
| --- | --- | --- | --- | --- | --- | --- | --- |
|  | *N*=81 (%)  *n* (%) | | *N*=1,012 (%)  *n* (%) | | *N*=1,093 (%)  *n* (%) | |  |
| **Symptomatic (inguinal lymph node enlargement, ulceration in exogenitalia, purulent urethral discharge)** | | | | | |  | 0.268^‡^ |
| Yes | 12/77 | (15.6) | 105/925 | (11.4) | 117/1001 | (11.7) |  |
| inguinal lymph node enlargement | 2/12 | (16.7) | 12/103 | (11.7) | 14/115 | (12.2) | 0.608^‡^ |
| ulceration in exogenitalia | 3/12 | (25.0) | 40/103 | (38.8) | 43/115 | (37.4) | 0.375^‡^ |
| purulent urethral discharge | 9/12 | (75.0) | 53/103 | (51.5) | 62/115 | (53.9) | 0.135^‡^ |
| **Other STD (syphilis, gonorrhea, condyloma)** | | | |  |  |  | 0.225^‡^ |
| Yes | 19/77 | (24.7) | 172/926 | (18.6) | 191/1003 | (19.0) |  |
| Syphilis | 6/19 | (31.6) | 26/171 | (15.2) | 32/190 | (16.8) | 0.098^‡^ |
| Gonorrhea | 4/19 | (21.1) | 38/171 | (22.2) | 42/190 | (22.1) | 0.943^‡^ |
| Condyloma | 2/19 | (10.5) | 42/171 | (24.6) | 44/190 | (23.2) | 0.173^‡^ |
| Unknown | 7/19 | (36.8) | 65/171 | (38.0) | 72/190 | (37.9) | 0.921 |
| **STD treatment completed** | |  |  |  |  |  | <0.001 |
| Yes | 17/19 | (89.5) | 158/188 | (79.4) | 175/207 | (84.5) |  |
| **Syphilis test** |  |  |  |  |  |  | <0.001^‡^ |
| Positive | 16/81 | (19.8) | 67/1012 | (6.6) | 83/1093 | (7.6) |  |

^〒^. Pearson Chi-Square. ^‡^. Fisher's Exact Test
