# Supplementary material for: Risk Factors for HIV-1 seroconversion among Taiwanese men visiting gay saunas who have sex with men
Source: BMC Infect Dis. 2011 Dec 5;11:334. doi: 10.1186/1471-2334-11-334 (PMC3295735; doi:10.1186/1471-2334-11-334)
Supplement: Additional file 6 — Phylogenetic analysis of HIV-1 strains among Taiwanese men having sex with men (MSM) who visited gay saunas. The maximum-likelihood tree was constructed with the MEGA program (vers. 5.0) using the env nucleotide sequence from different HIV-1 strains. Bootstrap values (1000 bootstrap samples) are indicated beside the branches in percent. HIV-1 strains from the gay saunas (circle symbol) and the Venereal Disease Control Institute (VDCI) (triangle symbol) were labeled and characterized to denote the patients' characteristics, including the year of diagnosis, from 2001 to 2003; the sex of the patient (M, male); the risk factor of the patient (Ho, homosexual; He, heterosexual; Bi, bisexual); and the location of the gay saunas (N, north; M, central). The scale bar indicates the evolutionary distance. [file 1471-2334-11-334-S6.DOC]

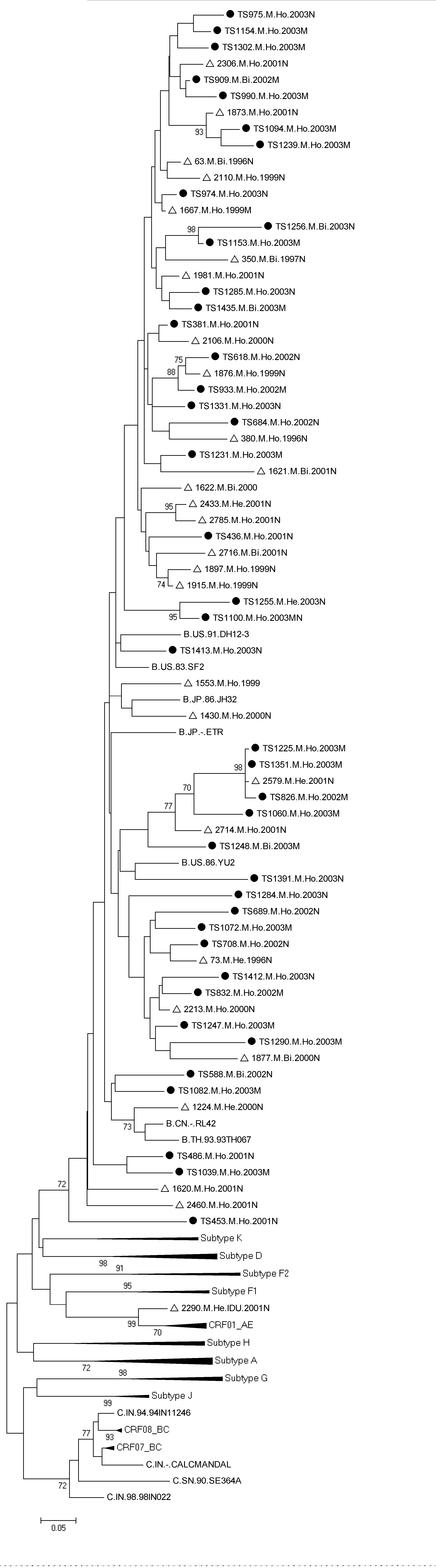


**Additional file 6 - Phylogenetic analysis of HIV-1 strains among Taiwanese men having sex with men (MSM) who visited gay saunas.** The maximum-likelihood tree was constructed with the MEGA program (vers. 5.0) using the *env* nucleotide sequence from different HIV-1 strains. Bootstrap values (1000 bootstrap samples) are indicated beside the branches in percent. HIV-1 strains from the gay saunas (circle symbol) and the Venereal Disease Control Institute (VDCI) (triangle symbol) were labeled and characterized to denote the patients’ characteristics, including the year of diagnosis, from 2001 to 2003; the sex of the patient (M, male); the risk factor of the patient (Ho, homosexual; He, heterosexual; Bi, bisexual); and the location of the gay saunas (N, north; M, central). The scale bar indicates the evolutionary distance.
